# Bumble Bees (Hymenoptera: Apidae: *Bombus* spp.) of Interior Alaska: Species Composition, Distribution, Seasonal Biology, and Parasites

**DOI:** 10.3897/BDJ.3.e5085

**Published:** 2015-05-08

**Authors:** Rehanon Pampell, Derek Sikes, Alberto Pantoja, Patricia Holloway, Charles Knight, Richard Ranft

**Affiliations:** ‡ United States Department of Agriculture, Agricultural Research Service, Subarctic Agricultural Research Unit, AK, Fairbanks, United States of America; § University of Alaska Museum, Fairbanks, United States of America; | University of Alaska Fairbanks, School of Natural resources and Agricultural Sciences, Fairbanks, United States of America; ¶ State of Alaska, Department of Natural Resources, Division of Agriculture, Fairbanks, United States of America; # United States Department of Agriculture, Agricultural Research Service, Subarctic Agricultural Research Unit, Fairbanks, United States of America

**Keywords:** Bumble bees, *
Bombus
*, Alaska, Hymenoptera, diversity, subarctic

## Abstract

**Background:**

Despite the ecological and agricultural significance of bumble bees in Alaska, very little is known and published about this important group at the regional level. The objectives of this study were to provide baseline data on species composition, distribution, seasonal biology, and parasites of the genus *Bombus* at three major agricultural locations within Alaska: Fairbanks, Delta Junction, and Palmer, to lay the groundwork for future research on bumble bee pollination in Alaska.

**New information:**

A total of 8,250 bumble bees representing 18 species was collected from agricultural settings near Delta Junction, Fairbanks, and Palmer, Alaska in 2009 and 2010. Of the 8,250 specimens, 51% were queens, 32.7% were workers, and 16.2% were males. The species composition and relative abundances varied among sites and years. Delta Junction had the highest relative abundance of bumble bees, representing 51.6% of the specimens collected; the other two locations, Fairbanks and Palmer represented 26.5% and 21.8% of the overall catch respectively. The species collected were: *Bombus bohemicus* Seidl 1837 (= *B. ashtoni* (Cresson 1864)), *B. balteatus* Dahlbom 1832, *B. bifarius* Cresson 1878, *B. centralis* Cresson 1864, *B. cryptarum* (Fabricius 1775) (=*B. moderatus* Cresson 1863), *B. distinguendus* Morawitz 1869, *B. flavidus* Eversmann 1852 (=*B. fernaldae* Franklin 1911), *B. flavifrons* Cresson 1863, *B. frigidus* Smith 1854, *B. insularis* (Smith 1861), *B. jonellus* (Kirby 1802), *B. melanopygus* Nylander 1848, *B. mixtus* Cresson 1878, *B. neoboreus* Sladen 1919, *B. occidentalis* Greene 1858, *B. perplexus* Cresson 1863, *B. rufocinctus* Cresson 1863, and *B. sylvicola* Kirby 1837. Overall, the most common bumble bees near agricultural lands were *B. centralis*, *B. frigidus*, *B. jonellus*, *B. melanopygus*, *B. mixtus*, and *B. occidentalis*. Species' relative population densities and local diversity were highly variable from year to year. *Bombus occidentalis*, believed to be in decline in the Pacific Northwest states, represented 10.4% of the overall specimens collected from the three sites studied. Bumble bees were found to be infected by *Nosema* and nematodes with infection rates up to 2.1% and 16.7% respectively. Of the eight species infected by parasites, *B. occidentalis* displayed the highest *Nosema* infection, while *B. centralis* was the species with the highest infection of nematodes. To our knowledge this represents the first multi-year study on bumble bees from the main agricultural areas of Alaska to provide baseline data on species composition, distribution, seasonal biology, and parasites of the genus *Bombus*.

## Introduction

Bumble bees are considered important pollinators in subarctic Alaska (Washburn 1963, Kevan 1972). Bumble bees will forage during rainy, cool, and windy weather during which honey bee activity is limited (Buchmann 1983). They have even been observed foraging during snowfall, under a full moon (Kearns and Thomson 2001), during the night, above the tree line (Richards 1973, Lundberg 1980), and in temperatures as cold as -3.6°C (Heinrich 1979). Native bees, such as bumble bees, are responsible for the pollination of over $3 billion US dollars worth of fruits and vegetables produced in the US (Losey and Vaughan 2006).

Despite the ecological importance of bumble bees, no published estimates on the value of bumble bee pollination for crops in Alaska are available. Furthermore, there is no consensus on the total number of *Bombus* species present in Alaska with estimates ranging from 17-24 species (Washburn 1963, Krombein et al. 1979, Bishop and Armbruster 1999, UAM 2013b). These estimates suggest the *Bombus* fauna of Alaska contains about half of the species known for North America. Williams et al. (2014), the most recent and authoritative publication on North American *Bombus*, list 23 species from Alaska.

Nationwide, honey bees are undergoing extensive die-offs which do not appear to have a single underlying cause; a phenomenon termed Colony Collapse Disorder (CCD) (Bromenshenk et al. 2010). Some predict that native bees will buffer potential declines in agricultural production due to CCD (Buchmann and Nabhan 1996, Kremen 2005, Kremen and Ostfeld 2005, Winfree et al. 2007), but in many cases, as in Alaska, the native bee fauna is poorly known. There are also concerns about the long-term persistence of bees, some of which are predicted to become extinct, as a result of the planet's changing climate (Rasmont et al. 2015).

*Nosema* is a genus of obligate microsporidian intracellular parasites that has been known to affect economically important insects such as the silkworm moth, honey bees, and bumble bees (Otti and Schmid-Hempel 2007, Koch and Strange 2012). *Nosema bombi* infestation has been related to declining bumble bee populations and reduced genetic diversity of North American bumble bees (Flanders et al. 2003, Thorp and Shepherd 2005, Colla et al. 2006, Cameron et al. 2011).

Impoverished native bumble bee communities are often associated with the intensification of agriculture and may be insufficient to replace the pollination services currently provided by honey bees (Goulson et al. 2008). Alaskan farms tend to be surrounded by native vegetation and habitat that would benefit native bee populations, but there is little information on bumble bee species composition, geographical distribution, biology, and factors affecting bumble bee species richness associated with agricultural areas in the state. The objectives of this study were to provide baseline data on species composition, distribution, seasonal biology, and parasites of the genus *Bombus* at three agricultural locations within Alaska: Fairbanks, Delta Junction, and Palmer.

## Material and methods

Stephen and Rao (2005)The three major agricultural areas of Alaska (Benz et al. 2009) with farms were sampled in 2009 and 2010. These were the University of Alaska Fairbanks experimental farms near Delta Junction (64.04°N, 145.73°W), Fairbanks (64.85°N, 147.85°W), and Palmer (61.60°N, 149.13°W, WGS84). Habitat types surrounding field sites ranged from urban areas with mixed boreal forest and a botanical garden near Fairbanks, grasslands and boreal forest near Delta Junction, and large scale commercial agricultural lands near urban areas in Palmer. All three locations grow potatoes, barley, wheat, oats, oilseeds (camelina, canola, and mustard), and rhubarb.

Blue vane Japanese beetle traps (SpingStar Inc; Woodinville, Washington) were placed (five traps per site per year) around agricultural field perimeters and set at a height of one meter from ground level following the methods described by Stephen and Rao (2005). In our study, traps were hung horizontally to prevent rain from entering traps. Both years, the traps were placed along a tree or fence line 200 meters apart in a straight line along the same field edge. Traps had a 6.5 cm^2^ piece of Vaportape ® (Hercon Environmental; Emigsville, Pennsylvania) in the bucket to kill captured insects. The vaportape was replaced every 6 weeks. Traps were serviced every seven days; bumble bees were removed, transported to the laboratory, and stored in labeled Ziploc® bags, and frozen until they could be pinned, labeled and identified in the Agricultural Research Service (ARS) laboratory in Fairbanks, Alaska. Sampling dates were May 19 to September 10, 2009 and March 27 to September 28, 2010 in Delta Junction; March 27 to September 23, 2009 and May 3 to September 27, 2010 in Fairbanks; May 4 to September 21, 2009 and May 17 to October 7, 2010 in Palmer.

Initially, a series of Alaskan specimens were identified by Dr. Jamie Strange, United States Department of Agriculture (USDA), Agricultural Research Service (ARS), Pollinating Insects Research Unit, Logan, Utah. Subsequent identifications were made using the keys of Thorp et al. (1983) and Stephen (1957) as well as comparison to the voucher collection identified by Strange. Voucher specimens were deposited in the University of Alaska Museum (UAM) Insect Collection, Fairbanks, Alaska. Records of these specimens are available online via the UAM database (UAM 2013a).

A series of taxonomic changes resulting from recent DNA barcoding work (Cameron et al. 2007, Williams et al. 2012) were made subsequent to the start of, but prior to the completion of, this project. As a result, some species in our study were referenced (on specimen labels, lab notes, data files, etc.) using now invalid names. In this paper we have used the currently valid names from Williams (1988) and Williams et al. (2012).

Two trials were conducted to establish the presence of entomoparasites in bumble bees. From May 26 to September 17, 2010, ten bees per week were hand collected from the University of Alaska Fairbanks, Georgeson Botanical Garden (GBG) and frozen until their abdomens were dissected following the procedure described by Klee et al. (2006) and Plischuk et al. (2009). Bumble bees were collected with the aid of a glass jar. Only bees resting on flowers or structures were collected. During 2011, the sampling technique was modified and bees were captured with blue vane traps as previously described. Ten traps per locality (Fairbanks and Palmer) were setup for 24hr periods once a week from 3 May - 16 September 2011. Dissected digestive/reproductive tracts were homogenized in 2 ml of distilled water and the homogenate examined by light microscopy (400x) to determine the presence of microsporidian-like spores of Nosema (Klee et al. 2006, Plischuk et al. 2009).

Nematodes were observed while looking for *Nosema*. The nematodes were placed on baby food plates for nematodes according to the methods of Stock et al. (2001). Nematodes were identified by Patricia Stock, University of Arizona Department of Entomology. The percentage of bumble bees infested by *Nosema*-like spores and nematodes was calculated.

## Data resources

Delta Junction data set: http://arctos.database.museum/saved/USDA-Bombus-Delta

Fairbanks data set: http://arctos.database.museum/saved/USDA-Bombus-Fairbanks

Palmer data set: http://arctos.database.museum/saved/USDA-Bombus-Palmer

From University of Alaska Museum Insect Collection, Arctos: http://dx.doi.org/doi:10.7299/X75D8S0H

## Results

Delta Junction had the highest relative abundance of bumble bees with 4,258 specimens representing 51.6% of the overall catch. Fairbanks and Palmer represented 26.5% and 21.8% of the overall catch respectively. Sixteen of the identified 18 species were collected from Delta Junction, while 14 species were identified from Fairbanks and Palmer. Of the 8,250 specimens examined, 51.0% were queens, 32.7% were workers, and 16.2% were males. Six of the 18 species collected in this study were found at all three locations during both sampling years: *B. centralis*, *B. frigidus*, *B. jonellus*, *B. melanopygus*, *B. mixtus*, and *B. occidentalis*.

**Delta Junction**

Sixteen species were collected from Delta Junction (Table 1). The most abundant species both years was *B. bifarius* representing approximately 46% and 54% of the specimens collected in 2009 and 2010 respectively. In 2009, three species, *B. bifarius* (46.3%), *B. jonellus* (17.1%), and *B. frigidus* (11.0%) represented 74% of the total bumble bees collected. In 2010, a different set of species, *B. bifarius*, *B. occidentalis*, and *B. jonellus*, contributed 76.4% of the specimens that year with percentages of 54.1, 12.4, and 9.9 respectively. Relative abundances were lower in 2010 (n = 2745 specimens) as compared to 2009 (n = 4020); however, the percentage of queens was higher in 2010 (79.1%) as compared to 2009 (47.5%).

**Figure 1.**
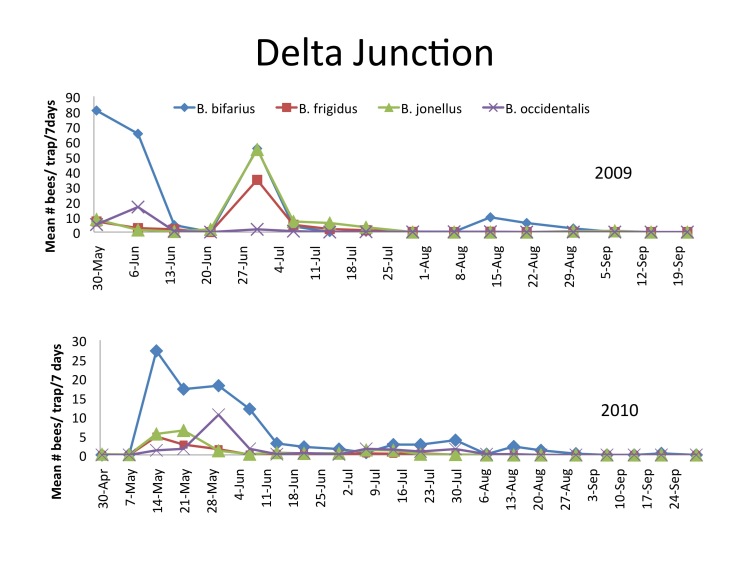
Mean number and standard errors of *B. bifarius*, *B. frigidus*, *B. jonellus*, and *B. occidentalis* per trap per 7 day sampling period collected with blue vane traps near Delta Junction, Alaska 2009 and 2010 (see Suppl. materials 1, 2)

Flight activity, represented by the mean number of bumble bees per trap per week during 2009 and 2010 is presented in Fig. 1 for the four most abundant species. The highest density recorded was 80.6 for *B. bifarius* per trap per week (May 30, 2009). This species, was also the most abundant species in 2010, but relative density was almost half of that recorded in 2009 with a maximum density of 27.2 bees per trap per week (May 14, 2010). With the exception of *B. bifarius* in August 2009, densities remained below 10 bees per week after mid-June during both years. The western bumblebee, *B. occidentalis*, was present in both years reaching a maximum density of 10.5 bees per trap per week in early June 2009.

**Fairbanks**

Fifteen species were collected from Fairbanks during the 2009 season (Table 2). Ninety seven percent of the specimens were collected in 2009 and 3% in 2010. Only seven species, *B. centralis*, *B. frigidus*, *B. jonellus*, *B. melanopygus*, *B. mixtus*, *B. occidentalis*, *B. perplexus* were collected during 2010.

**Figure 2.**
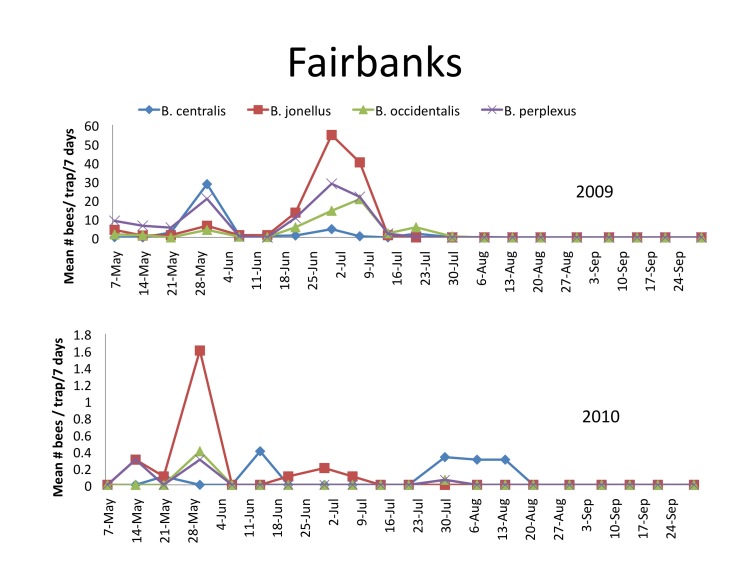
Mean number and standard errors of *B. centralis*, *B. jonellus*, *B. occidentalis*, and *B. perplexus* per trap per 7 day sampling period collected with blue vane traps near Fairbanks, Alaska 2009 and 2010. (see Suppl. materials 3, 4).

Flight activity was earlier in 2010 than in 2009 (Fig. 2), but relative abundance was lower than 2009 (Table 2). The four most abundant species were the same in both years, but the relative density of *B. jonellus* was higher in 2010 (42.1%) than in 2009 (29.9%). The highest relative abundance recorded in Fairbanks was 54.6 and 1.6 bees per trap week for *B. jonellus* on June 30, 2009 and May 30, 2010 respectively. Neither *B. perplexus* nor *B. jonellus* were collected after July 21; however *B. occidentalis* displayed flight activity until July 30, 2009. All flight activity ended by July 30 in 2009 and August 21 in 2010.

**Palmer**

Fourteen species were collected from Palmer (Table 3). All species collected from Palmer were previously recorded from the other two collecting sites. Not all species were present both years in Palmer; *B. sylvicola* was collected in low numbers in 2009, but not recovered in 2010. On the other hand, *B. bifarius*, which was collected in low numbers in 2010, was not collected during the 2009 season. The most abundant species both years was *B. centralis* representing 40% and 36.6% for 2009 and 2010 respectively.

**Figure 3.**
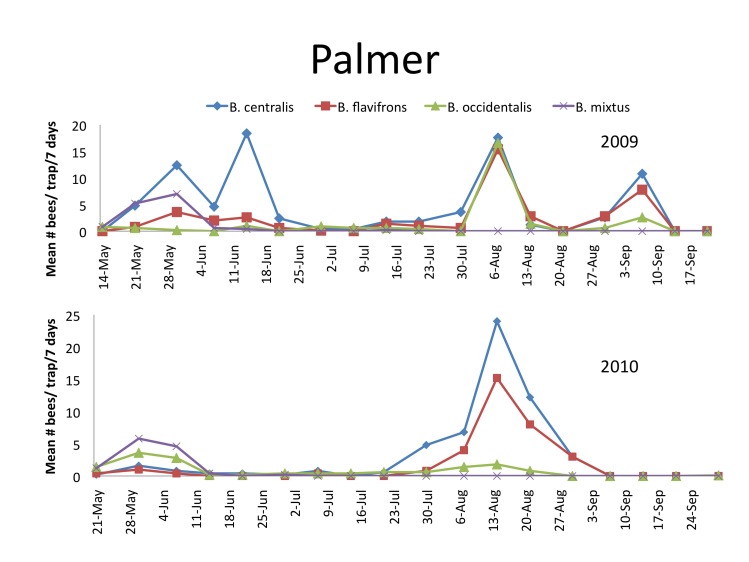
Mean number and standard errors of *B. centralis*, *B. flavifrons*, and *B. occidentalis* per trap per 7 day sampling period collected with blue vane traps near Palmer, Alaska 2009 and 2010 (see Suppl. materials 5, 6).

In 2009, three species, *B. centralis*, *B. flavifrons*, and *B. occidentalis*, contributed 71.5% of the specimens with 40%, 20%, and 13.1 % respectively (Table 3, Fig. 3). In 2010 the same three species contributed 69.1% of the specimens that year, but the relative abundance differed with 36.6%, 22.32%, and 10.1 % for *B. centralis*, *B. flavifrons*, and *B. occidentalis*, respectively (Table 3, Fig. 3). The fourth species in order of abundance was *B. mixtus*, but relative densities were low most of the season during both years.

In 2009, *B. centralis* and *B. occidentalis* were collected as early as 14 May (Fig. 3). One of the species, *B. centralis*, displayed four peaks of flight activity on May 30, June 14, August 7 and September 7, 2009. The highest density collected in this location was for *B. centralis* during the month of August with a mean of 20 bees per trap per week on August 14, 2010 and 17.6 bees per trap per week on August 7, 2009. No bees were collected after September 14 of any year.

**Parasites**

Nematodes were found in nine specimens of two species among 101 *Bombus* specimens examined. Infection incidence was 16.7% of *B. centralis* specimens and 6.3% of *B. perplexus* specimens. The nematodes were identified as belonging to the family Tetradonematidae.

A total of 642 bumble bee specimens, from Fairbanks and Palmer, of seven species were examined for microsporidians (*Nosema* spp.) (Table 4). Microsporidian infection varied by species and sites. Only two of the species examined, *B. occidentalis* and *B. flavidus* (=*B. fernaldae*), displayed infection in both localities, but all infections were below 1%. The highest incidence of *Nosema* was detected in *B. occidentalis* with 2.1% of Palmer specimens infected and 1.2 % of Fairbanks specimens infected.

## Discussion

All the species recovered have been previously reported from Alaska in collections, databases, and publications. However, to our knowledge, this represents the first multi-year study focused on seasonality and abundance of Alaskan Bombus species in the major agricultural regions of the state. This also represents the first report on nematodes and the second report on *Nosema* affecting bumble bees in Alaska.

Although no published reports are available on bumble bee population dynamics in Alaska's major agricultural areas to compare with our results, working with other taxa, Pantoja et al. (Pantoja et al. 2009, Pantoja et al. 2010b, Pantoja et al. 2010a) reported a similar pattern of higher relative densities of leafhoppers (Cicadellidae) and wireworms (Elateridae) in Delta Junction relative to Fairbanks and Palmer. Pantoja et al. (Pantoja et al. 2009, Pantoja et al. 2010a, Pantoja et al. 2010b) suggested that the differences in relative leafhopper and wireworm densities were associated with climatic differences, cropping histories, habitat availability, or agronomic practices. Bumble bee diversity and abundance can be affected by the availability of floral resources and nest sites, climatic conditions, presence of invasive species, habitat fragmentation, parasitic spillover, urbanization, competition, and the use of pesticides (Cane and Tepedino 2001,Roubik 2001, Goulson et al. 2008). The three sites studied have significant climatic differences (Benz et al. 2009) and cropping histories (Pantoja et al. 2009) that might have affected relative bumble bee densities. Proximity to urban areas might provide another explanation for the differences in species composition and densities between sites. Traps in Delta Junction were located in rural areas, while traps in Fairbanks and Palmer were within two kilometers of major highways and structures. Bumble bee populations respond positively to the presence of unmanaged areas (pastures, meadows, and forests) that provide nesting and forage sites (Williams 1986). Davros et al. (2006), working with similar Conservation Reserve Program habitats, reported that butterfly densities and species are affected by habitat fragmentation. Delta Junction has large areas devoted to the Conservation Reserve Program with minimum disturbance (Seefeldt et al. 2010) that might have contributed to the higher bee densities there.

Eight species previously reported in Alaska, but not collected during our study are: *B. appositus* Cresson 1878, (possible misidentification of *B. borealis* Kirby 1837 or *B. distinguendus*), *B. fervidus* (Fabricius 1798), *B. hyperboreus* Schönherr 1809, *B. lucorum* Linnaeus 1761 (likely misidentification of *B. cryptarum*), *B. nevadensis* Cresson 1874, *B. sandersoni* Franklin 1913 (likely misidentification of *B. jonellus* (Kirby)), *B. sitkensis* Nylander 1848, and *B. vagans* Smith 1854 (Ashmead 1902, Bequaert 1920, Washburn 1963, Milliron 1973, Williams and Batzli 1982, Thorp et al. 1983, Henrich and Vogt 1993, Williams and Thomas 2005, Ascher and Pickering 2012, CNC (Canadian National Collection) 2010, UAM 2013a,Williams et al. 2014). These eight species represent possible misidentifications (e.g. *B. sandersoni*), rare interceptions outside the species' normal range (e.g. *B. fervidus*), or are rare species in Alaska (e.g. *B. vagans*, *B. sitkensis*).

Published literature on parasites of bumble bees in Alaska is scant. Schmid-Hempel and Tognazzo (2010) described a protozoan flagellate, *Crithidia bombi* Lipa and Triggiani in Alaskan bumble bees. Koch and Strange (2012), discuss the distribution and relative abundance of eight *Bombus* species in Alaska and the prevalence of *Nosema bombi* Fantham and Porter 1914, detected in *B. occidentalis*, *B. cryptarum* (as *B. moderatus*), *B. bifarius*, *B. flavifrons*, *B. jonellus*, *B. mixtus*, and *B. sylvicola*. To our knowledge, the discovery of nematodes in bumble bees from Fairbanks and Palmer represents the first report of this endoparasite from the state (Table 4).

The origin of the endoparasites observed is unknown. In Ontario, Canada, higher *Nosema* prevalence has been associated with commerically raised bumble bees that escaped greenhouses, a phenomenon known as "pathogen spillover" (Colla et al. 2006). However, Plischuk et al. (2009) reported that bumble bees can become infected by *Nosema* from honey bees. Due to climatic conditions, Alaskan beekeepers have been importing honeybees into Alaska annually since the early days of beekeeping in the state (Washburn 1974) which may have introduced these parasites to the state. It is possible that *Nosema* occurred naturally in Alaska but without historical data predating beekeeping in Alaska, this is uncertain.

Both parasites, *Nosema* and nematodes, were identified from bee species collected in high numbers from Fairbanks and Palmer. However, few specimens of the relatively low abundance species were examined for endoparasites. In the nematode study in Fairbanks, we only examined bees hand-collected while resting; this may have skewed the results towards bees in poor health. Research is also needed to study the geographical extent of nematodes and *Nosema* infecting bumble bees in Alaska. Tetradonematid nematodes are obligate and fairly specific parasites, but are not considered common nematodes of bumble bees (Poinar 1975), stressing the need to further study this group in Alaska.

The western bumble bee, *B. occidentalis*, once considered to be one of the most common North American west coast bumble bee species, is declining in the Pacific North West (Rao and Stephen 2007, Colla and Ratti 2010, Cameron et al. 2011). In Alaska, this species represented roughly 10.4% of the total specimens collected (Tables 1, 2, 3) suggesting that *B. occidentalis* is a relatively abundant species in the areas studied. Among all *Bombus* specimens in the University of Alaska Museum (n=23,001), this species is the fourth most abundant behind (in order of abundance) *B. bifarius*, *B. centralis*, and *B. jonellus*. Koch and Strange (2012) also noted that *B. occidentalis* appears to be both widely distributed and relatively common species in Alaska. This species comprised 28% of the bumble bees in their survey, which included a total of 15 *Bombus* species. In addition to the sites studied, this species was collected from the Kenai Peninsula near Soldotna and Wiseman, Alaska (data not presented but specimens vouchered in UAM, and data available online [UAM 2013b]). However, *B. occidentalis* had the highest *Nosema* counts among the six species in which we detected this parasite (Table 4). Koch and Strange (2012) also found Alaskan *B. occidentalis* to have the highest infection incidence (44%) among the seven species in which they found *Nosema*. Several authors (Whittington and Winston. 2004, Thorp 2005, Thorp and Shepherd 2005) have proposed that the recent catastrophic decline throughout North America of *B. occidentalis* was due to *Nosema*. Social parasites of *B. occidentalis* include *B. suckleyi*, *B. insularis*, and *B. flavidus* (=*B. fernaldae*) (Thorp et al. 1983), all of which occur in Alaska (Washburn 1963) and two species were documented in this survey; emphasizing the need to study the effect of social parasites on bumble bees in the state.

The earliest sampling date recorded was May 6; however, depending on sites and years, flight activity was detected during the first week sampling was initiated, suggesting that flight activity started before the snow melts. In this study we deployed traps as soon as snow melted; future studies should initiate sampling by mid-April, before the snow starts to melt.

The highest counts observed were in Delta Junction during 2009 with a mean number of 11.5 bumble bees per trap per day. No previous reports from Alaska provide comparative data to put these values in context. In Oregon, Stephen and Rao (2005) captured an average of 17.3 bees per day using the same blue vane trapping method.

Counts in Palmer were consistent between years with a difference of 276 bees between the two years (Table 3). The relatively stable densities between years in Palmer can be explained by trap locations. During both years, traps were hung in close proximity to a patch of *Rheum* spp. that provided a long and consistent foraging source for bees. Information on the *Rheum* species available at the site, flowering patterns, and other plant characters were discussed by Pantoja and Kuhl (2009).

Depending on site and year, queens were the most abundant caste collected. The lowest collecting year was 2010 and the location with the fewest queens was Fairbanks where only 57 specimens were collected. Delta Junction displayed the highest overall queen density. It is reasonable to assume that the removal of queens during the previous season (2009) would reduce the overall bumble bee relative density during the following season (2010). However, this was not observed, more queens were captured in Delta Junction during 2010 than 2009 (Table 2). The first year, sampling started when snow melted. We expect queens to emerge during drier conditions; however, to our surprise, queens were already emerging while snow was still present. In 2010, we set up traps a month earlier than the previous season and had several weeks of no activity before we began to see queens in the traps. The reduction in the relative population density of workers and males recorded for Delta Junction during the 2010 season as compared to the 2009 season cannot be explained by sampling or removal of the queens alone. In Oregon, Stephen and Rao (2005) did not distinguish between castes, but report collecting 70.1% females during their study.

Specimens collected in low densities (less than 15 specimens collected) include *B. balteatus*, *B. distinguendus*, and *B. neoboreus*. Little is known about these species in Alaska. Previous reports (Washburn 1963) provided limited or no information on these species, and lacked data on species' relative densities at each site or year.

One of the species collected in relatively high densities, *B. flavifrons*, has been identified as a primary pollinator of lingonberries, *Vaccinium vitis-idaea* Linnaeus 1753, (Davis 2002, Davis et al. 2003). This species was collected from Fairbanks and Palmer, but not from Delta Junction. Additional studies are needed to determine associations of bumble bee species with plants available in each geographic area of the state. Four of the six most common species in our study, *B. frigidus*, *B. centralis*, *B. jonellus*, *B. mixtus*, were identified as pollinators of bog blueberry, *Vaccinium uliginosum* L. Buxbaum (2011).

Three, and possibly a fourth species, of the 18 species collected belong to the subgenus *Psithyrus* Lepeletier de Saint-Fargeau 1832, which includes the cuckoo bumble bees (social parasites) *B. bohemicus* (=*B. ashtoni*), *B. flavidus* (=*B. fernaldae*), and *B. insularis*, (and possibly *B. suckleyi*). Two of the cuckoo bumble bees, *B. flavidus* (=*B. fernaldae*) and *B. insularis*, were recovered from the three sites surveyed; while *B. bohemicus* (=*B. ashtoni*), was not recorded from Fairbanks. The presence of *B. suckleyi* reported in Alaska by Washburn (1963) was not confirmed from our work, tentatively. This species is hard to distinguish from *B. bohemicus* (=*B. ashtoni*), one specimen (UAM:Ento:181710) keyed to *B. suckleyi* by J. Koch with some uncertainty. With the exception of the Palmer location, relative densities of cuckoo bumble bees were below six percent. Research is needed to better understand the effects of social parasites on bumble bee species in the state and their interaction with endoparasites like *Nosema* and nematodes.

This report provides baseline data on species composition, distribution, seasonality, and parasites of the genus *Bombus* at the main agricultural areas in Alaska: Fairbanks, Delta Junction, and Palmer. Baseline data are needed to help understand reported patterns of bumble bee declines in North America (Cameron et al. 2011). Additional research is needed to better understand the biology, geographical distribution, contribution of bumble bees to Alaska agriculture, and the possible effects of endo- and social parasites on bumble bees in the state.

## Supplementary Material

Supplementary material 1 Bombus spp trapped in Delta Junction Alaska, 2009 Data type: occurences
Brief description: 2,446 specimens of sixteen species trapped using Blue Vane pollinator traps with counts of queens, workers, and males by date.
File: oo_41535.xlsx Rehanon Pampell, Alberto Pantoja, Derek S. Sikes, Patricia Holloway, Charles Knight and Richard Ranft

Supplementary material 2 Bombus spp trapped in Delta Junction Alaska, 2010 Data type: occurences
Brief description: 1812 specimens of sixteen species trapped using Blue Vane pollinator traps with counts of queens, workers, and males by date.
File: oo_41536.xlsx Rehanon Pampell, Alberto Pantoja, Derek S. Sikes, Patricia Holloway, Charles Knight and Richard Ranft

Supplementary material 3 Bombus spp trapped in Fairbanks Alaska, 2009 Data type: occurences
Brief description: 2,131 specimens of fifteen species trapped using Blue Vane pollinator traps with counts of queens, workers, and males by date.
File: oo_41539.xlsx Rehanon Pampell, Alberto Pantoja, Derek S. Sikes, Patricia Holloway, Charles Knight and Richard Ranft

Supplementary material 4 Bombus spp trapped in Fairbanks Alaska, 2010 Data type: occurences
Brief description: 57 specimens of seven species trapped using Blue Vane pollinator traps with counts of queens, workers, and males by date.
File: oo_41540.xlsx Rehanon Pampell, Alberto Pantoja, Derek S. Sikes, Patricia Holloway, Charles Knight and Richard Ranft

Supplementary material 5 Bombus spp trapped in Palmer Alaska, 2009 Data type: occurences
Brief description: 1040 specimens of fourteen species trapped using Blue Vane pollinator traps with counts of queens, workers, and males by date.
File: oo_41541.xlsx Rehanon Pampell, Alberto Pantoja, Derek S. Sikes, Patricia Holloway, Charles Knight and Richard Ranft

Supplementary material 6 Bombus spp trapped in Palmer Alaska, 2010 Data type: occurences
Brief description: 764 specimens of fourteen species trapped using Blue Vane pollinator traps with counts of queens, workers, and males by date.
File: oo_41542.xlsx Rehanon Pampell, Alberto Pantoja, Derek S. Sikes, Patricia Holloway, Charles Knight and Richard Ranft

## Figures and Tables

**Table 1.** Sum (Suppl. materials 1, 2) of queens (Q), workers (W), males (M), and percentage of overall bumble bees collected with blue vane traps near Delta Junction, Alaska 2009-2010. Total specimen count for 2009 = 2,446; 2010 = 1812.

**Species**	**Author**	**2009**				**2010**			
		**Q**	**W**	**M**	**%**	**Q**	**W**	**M**	**%**
*B. bohemicus*	Seidl	8	0	0	0.6	2	0	0	0.1
*B. balteatus*	Dahlbom	4	1	0	0.2	3	0	0	0.2
*B. bifarius*	Cresson	739	315	90	46.3	794	138	49	54.1
*B. centralis*	Cresson	37	5	10	2.1	46	12	9	3.7
*B. cryptarum*	(Fabricius)	1	1	0	0.1	9	2	0	0.6
*B. flavidus*	Eversmann	6	0	0	0.3	4	0	1	0.3
*B. frigidus*	Smith	52	171	49	11	94	13	4	6.1
*B. insularis*	(Smith)	34	0	3	2.1	34	0	0	1.9
*B. jonellus*	(Kirby)	55	276	91	17.1	144	36	0	9.9
*B. melanopygus*	Nylander	12	60	47	4.8	57	5	3	3.6
*B. mixtus*	Cresson	101	30	3	5.4	73	2	0	4.1
*B. neoboreus*	Sladen	1	0	0		0	0	0	0
*B. occidentalis*	Greene	70	57	0	5.1	143	79	2	12.4
*B. perplexus*	Cresson	17	7	2	1.1	24	3	6	1.8
*B. rufocinctus*	Cresson	9	0	0	0.4	1	1	0	0.1
*B. sylvicola*	Kirby	15	31	36	3.3	8	2	11	1.2
**TOTAL**		**1161**	**954**	**331**		**1434**	**293**	**85**	

**Table 2.** Sum (Suppl. materials 3, 4) of queens (Q), workers (W), males (M) and percentage of overall bumble bees collected with blue vane traps near Fairbanks, Alaska 2009-2010. Total specimen count for 2009 = 2,131; 2010 = 57.

**Species**	**Author**	**2009**				**2010**			
		**Q**	**W**	**M**	**%**	**Q**	**W**	**M**	**%**
*B. bifarius*	Cresson	0	0	0	0	0	1	0	0.0
*B. centralis*	Cresson	170	27	3	9.4	5	8	3	28.1
*B. cryptarum*	(Fabricius)	0	9	0	0.4	0	0	0	0.0
*B. distinguendus*	Morawitz	0	1	0	0.1	0	0	0	0
*B. flavidus*	Eversmann	2	0	2	0.2	0	0	0	0.0
*B. flavifrons*	Cresson	0	0	3	0.1	0	0	0	0.0
*B. frigidus*	Smith	74	58	34	7.8	1	0	0	1.8
*B. insularis*	(Smith)	8	0	2	0.5	0	0	0	0.0
*B. jonellus*	(Kirby)	94	328	217	29.9	21	3	0	42.1
*B. melanopygus*	Nylander	79	110	33	10.4	1	0	0	1.8
*B. mixtus*	Cresson	20	0	0	0.9	2	0	0	3.5
*B. occidentalis*	Greene	42	246	0	13.5	4	1	0	8.8
*B. perplexus*	Cresson	253	306	6	26.5	6	1	0	12.3
*B. rufocinctus*	Cresson	1	0	0	0.1	0	0	0	0.0
*B. sylvicola*	Kirby	1	1	1	0.1	0	0	0	0.0
**TOTAL**		**744**	**1086**	**301**		**40**	**14**	**3**	

**Table 3.** Sum (Suppl. materials 5, 6) of queens (Q), workers (W), males (M) and percentage of overall bumble bees collected with blue vane traps near Palmer, Alaska 2009-2010. *One specimen (UAM:Ento:181710) keyed to *B. suckleyi* by J. Koch with some uncertainty. Total specimen count for 2009 = 1,040; 2010 = 764.

**Species**	**Author**	**2009**				**2010**			
		**Q**	**W**	**M**	**%**	**Q**	**W**	**M**	**%**
B. bohemicus*	Seidl	0	0	0	0.0	54	0	0	7.1
*B. balteatus*	Dahlbom	1	0	0	0.1	0	1	0	0.1
*B. bifarius*	Cresson	0	0	0	0.0	1	0	0	0.1
*B. centralis*	Cresson	227	49	140	40.0	35	160	85	36.6
*B. cryptarum*	(Fabricius)	0	0	1	0.1	2	0	0	0.2
*B. flavidus*	Eversmann	16	0	3	1.8	1	0	0	0.1
*B. flavifrons*	Cresson	52	15	141	20.0	14	60	96	22.3
*B. frigidus*	Smith	28	5	2	3.4	11	3	0	1.8
*B. insularis*	(Smith)	22	0	4	2.5	63	0	0	8.3
*B. jonellus*	(Kirby)	25	4	1	2.9	32	0	1	4.3
*B. melanopygus*	Nylander	85	5	1	8.8	2	5	0	0.9
*B. mixtus*	Cresson	61	10	0	6.8	59	1	1	8.0
*B. occidentalis*	Greene	8	41	87	13.1	42	29	6	10.1
*B. sylvicola*	Kirby	6	0	0	0.6	0	0	0	0.0
**TOTAL**		**531**	**129**	**380**		**316**	**259**	**189**	

**Table 4.** Percentage of *Bombus* spp. infected with *Nosema*, Fairbanks and Palmer, Alaska, 2011. n = 402 specimens examined for Fairbanks, n = 240 specimens examined for Palmer.

**Species**	**Fairbanks %**	**Palmer %**
*B. bifarius*	0	0.42
*B. centralis*	0.48	0
*B. jonellus*	0.48	0
*B. flavidus*	0.48	0.42
*B. occidentalis*	1.2	2.1
*B. melanopygus*	0.48	0
*B. sylvicola*	0.48	0
TOTAL	2.5	2.9
